# Analysis of Stress and Displacement Fields in Prosthetic Crowns Made of Zirconium Dioxide Using Numerical Approach of Homogenization Hypothesis

**DOI:** 10.3390/ma15217716

**Published:** 2022-11-02

**Authors:** Michał Dzięgielewski, Kinga Regulska, Ryszard Korycki, Leszek Klimek

**Affiliations:** 1 Department of Mechanical Engineering, Informatics and Chemistry of Polymer Materials, Lodz University of Technology, Żeromskiego 116, 90-924 Lodz, Poland; 2 Interdisciplinary Doctoral School, Lodz University of Technology, Żeromskiego 116, 90-924 Lodz, Poland; 3 Institute of Materials Science and Engineering, Lodz University of Technology, Żeromskiego 116, 90-924 Lodz, Poland

**Keywords:** numerical modeling, analysis, prosthetic crowns, stress and displacement fields, zirconium dioxide, engineering model

## Abstract

The main goal of this paper is to analyze the stress and displacement fields in prosthetic crowns made of zirconium dioxide using the numerical approach of homogenization hypothesis. The simple engineering model is developed and applied in case of vertical forces. The model is a three-dimensional simulation of molars subjected to crushing, mastication, and clenching. Two basic approaches are considered: the single prosthetic crown on a single molar, and the prosthetic bridge on two molars. The distributions of material parameters are determined for the rigid support and the elastic gum structure of the homogenized properties. The crown on a single molar is analyzed in respect of caries, which are represented by weak material parameters. Irrespective of the problem, the maximal stresses are always insignificant compared to the compressive strength for enamel, dentin, periodontium, and zirconium dioxide. In case of caries, the maximal stresses are located at the contact surface caries/crown, whereas the displacement was higher than the same parameter without caries. The stresses inside the prosthetic bridge on two molars were comparable for elastic and rigid support, and located at the same areas. The molar displacement for elastic gum was higher than for the rigid base, and additionally supplemented by the displacement of the supporting structure.

## 1. Introduction

The main idea of all prosthetic restorations, for example, crowns, bridges, veneers, work on implants, etc., is to reconstruct the tooth tissue after the destruction of past injuries to, or cavities inside the enamel or dentin. Moreover, the permanent prosthetic restorations improve the external appearance and aesthetics of discolored teeth or their irregular shape; for example, after previous root-canal works.

Nowadays, aesthetic dentistry is becoming more and more popular. Patients often reach for permanent prosthetic restorations to improve their external appearance.

The preparation of a tooth for permanent prosthetic restoration requires grinding a large volume of the dentine which is still healthy. Before the prosthetic treatment, an endodontist’s intervention is recommended to heal the root canal inside the tooth, where the crown is next mounted. This means that the patient is required to heal a healthy tooth, but it causes the survival rate of the crown to increase significantly, and the risk of tooth decay under an artificial tooth is considerably reduced. The basic functions of the crown are the strengthening and protection of an abutment tooth.

Prosthetic crowns consist of a cup made from zirconium dioxide or metal alloys, such as chrome-cobalt and dental porcelain. The porcelain has a form of powder which, when mixed with liquid, becomes an aqueous suspension.

In traditional restorations where the cup was made from metal alloys, we needed to cover the cup using opaque paste. For cups made from zirconium dioxide, we do not need to do it. The light color of ZrO_2_ allows to use only dental veneering porcelain.

There are various types of porcelain designed for any type of material for prosthetic caps. Their chemical internal structure is different, as well as their compatibility with the cup material.

Making a prosthetic crown requires accuracy in mapping the structure of the prosthetic pillar, and is individually-oriented. The success of prosthetic treatment results from: (i) the pre-prosthetic procedure; (ii) the preparation of the abutment tooth; (iii) precisely mapping the tooth and adjacent tissues during the impression process; (iv) the accuracy of gypsum models and the quality of their formation [1,2]. Mistakes can appear at each stage of the above procedures and significantly determine the long-term use of artificial dentition, the reduced marginal tightness, and the placement on the abutment tooth [3,4]. The dentist, dental technician, and, above all, the patient’s commitment decide the success of the prosthetic treatment.

Prosthetic crowns are traditionally made using the lost-wax method. The final structure is cast on the basis of a previous wax model. The developed prosthetics and computer methods cause the CAD/CAM technology connected with milling to be more and more popular and frequent. The method is very precise: the edge tightness is of the order of 30 µm, compared to the generally acceptable tightness for the fixed prosthetic structures of 100 µm [5,6,7].

An ideal, all-ceramic dental material should be characterized by excellent aesthetic properties, such as translucency, transparency, color as close as possible to one’s own teeth, and the highest values of mechanical properties in order to be able to use it for many years. Until now, zirconium oxide was considered the best choice, and it has the lowest plaque accumulation value compared to other prosthetic restorations [6].

The new generations of ceramic materials have recently been introduced as materials for dental crowns; for example, zirconium dioxide, ZrO_2_. Nowadays, aesthetic dentistry is becoming more and more popular. Patients often reach for permanent prosthetic restorations to improve their external appearance. Zirconium oxide displaces the commonly used metal alloys [6,8]. The main problem of metal alloys is the opacity of the cup which precludes the aesthetic effective restoration of a permanent tooth. The gray tint is hard to cover with the veneering material. Moreover, the metal alloys can create allergies in the oral cavity manifested by bruising or swelling of the gums. Ions penetrate the tissues and cause this appearance. Allergy may occur because of corrosion also. The most common allergy is nickel allergy; that is why when making a restorations, we use cobalt-chrome alloy. It is estimated that 14% of women are allergic to nickel, and 2% of men. Cobalt-chrome alloys also have a small percentage (around 1%) of nickel in the structure. Allergies often cause the dentist to have to remove a restoration and later replace the prosthetic restoration for the new one, which involves additional costs. At present, there is no known allergy to zirconium dioxide [9].

The structures made of zirconium dioxide can be designed using CAD/CAM techniques. First, the three-dimensional images of teeth are created to determine the framework, and, next, the CAD model of the restoration. Thus, an intraoral scanner is generally available. Once the shape is accepted, the milling machine creates the prosthetic restoration from the ready-made zirconium blocks. The finished prosthetic cup is covered with ceramics in the form of a water suspension and then fired in a porcelain furnace. This means the structure of the material of a cup has to be properly prepared to get the best quality of connection between them [5,6]. Zirconium dioxide and veneering porcelain are connected on the basis of micromechanical coupling. The veneering materials do not have the sufficient mechanical properties compared to the zirconium suprastructure. Thus, a strong connection of both materials is the deciding factor [10].

The zirconium-based restorations in dental prosthetics transfer load up to 750 N [6,10]. The prosthetic zirconium dioxide ZrO_2_ is made of grains of the order of (0.2–0.3) µm and does not contain the glass phase. The material is very durable, resistant to abrasion, but brittle. The range of tensile strength is (900–1200) MPa, the crack strength is (2–6) MPa∙m^1/2^, and the shear strength is 2000 MPa. Zirconium dioxide comes in three structural phases: monoclinic, tetragonal, and cubic. Prosthetics use a tetragonal phase that does not have a glass phase in a structure. It is the most advantageous from the point of view of biomechanics, because it can be stabilized at room temperature by adding oxides of magnesium, calcium, yttrium, or cerium [6,11,12]. The material is available in the form of ready-made blocks, which are processed by a milling machine to the prosthetic framework during a sequence of consecutive steps [13].

Some works are devoted to numerical simulations of prosthetic structures. Bramanti et al. [14] underline the mechanical properties of dental prosthodontics crowns in order to differentiate the possibility of using each material for typical clinical conditions and masticatory load. The von Misesstress distributions are determined for different common dental crowns and materials using the FEM analysis and three-dimensional virtual model.

An individual patient’s case is often beyond the accepted treatments in implant dentistry compared with the crowns with larger overhang. In such cases, the stresses and their influence on implants are analyzed for different values of concentrated forces by Materac, Niesłony [15].

The stress distribution for three or four units of zirconium fixed partial dentures with three differences in connector thicknesses under occlusal forces are discussed in [16]. The results show that the stresses do not differ for the finite element (FE) models with different connector thickness. However, the stress values increased for the four-unit zirconium restorations than the three units.

The aims of the study [17] were to perform the 3-D computer simulation of mastication and clenching, analyze the pressure acting on the cusps of the mandibular molar, and to assess the stresses in the tooth tissues according to the modified von Mises criterion [17]. Computer simulations of clenching and the mastication of molars with three elastic modules, a carrot, an almond, and a nut, were performed. The maximum values of equivalent stresses were compared with the tensile strength of dentin and enamel. The computer simulation of the mastication cycle showed changes in the pressure distribution acting on the occlusal surfaces of molar and spatial stress in that tooth. The functional cusps were under the highest pressure towards the end of the closing phase of mastication and clenching. The value of stresses around the central groove in the enamel increased at the increase in elastic modulus of bolus.

Dejak [18] compared the strength of teeth that were restored with zirconia ceramic, leucite ceramic, composite resin, and acrylic crowns. The equivalent stresses in ceramic zirconia and leucite crowns were higher than those in resin composite and acrylic crowns, and the values did not exceed the tensile strength of these materials.

Pham et al. [19] evaluated the wear resistance of three-dimensional printed denture teeth resin compared to three commercially available prefabricated denture teeth. The three-dimensional printed denture teeth demonstrated better wear resistance compared to the commercially prefabricated denture teeth when opposed to zirconia.

The external surface of the prosthetic crown can be reinforced by other materials, e.g., the application of powders. Abbas and Rasheed [20] analyzed the copper-doped titanium dioxide (TiO_2_) nano-powder of different doping concentrations, fabricated with a classical method. The same authors introduced the references concerning the problem.

Khoury-Ribas et al. [21] performed free-style masticatory tests. Masticatory performance, masticatory laterality, and chewing rate were assessed.

Finite element (FE) modeling is commonly applied to determine the structural response to different loads in dental research. The distributions of stresses, strain, and displacements fields can be determined for each tooth structure and applied load. The authors introduce the FE modeling to specify the stresses and displacements of molars loaded by vertical force as a model of mastication and clenching. Two typical problems are considered: the crowns are located on the single molar, and the prosthetic bridge on two molars. The structures can be subjected to clenching and mastication, and the corresponding clenching force is considerably lower than the mastication load. Thus, the FE models of prosthetic crowns are subjected to alternative loads acting vertically as a model of compressive loads. The FE mesh is relatively difficult to create, and it needs some structural adaptations, which are a kind of optimization procedure. We consider two mesh systems, the tetragonal and the hexagonal, as well as the additional adaptive procedures such as the virtual topology, the smoothing procedures, etc. The additional problem is the molar support, which can be assumed as the rigid or elastic model of jaw movements during mastication and clenching. The correct implementation of FE requires to introduce the appropriate boundary and initial conditions for these problems. The material response is characterized by means of the stresses (i.e., von Mises and principal stress) and the displacement fields.

The main goal of this paper is to analyze the stresses and displacements fields in prosthetic crowns made of zirconium dioxide. The simple engineering model is developed and applied in case of vertical forces. The model introduces the behavior of a molar subjected to crushing, mastication, and clenching to analyze the pressure applied to its upper surface and to determine the stresses in the tooth according to the modified von Mises criterion. The strength of the tooth is of fundamental importance when describing the permissible load that is transmitted by the material, as well as the fatigue strength, i.e., the action distributed over time. The numerical procedure determines the material response of the crown, and is cheaper than laboratory tests. We introduce two basic approaches: the single prosthetic crown on a single molar, and theprosthetic bridge on two molars. The additional analysis concerns the single crown with caries represented by the weak material of the regular cylindrical form.

The novelty elements are the following: (i)the crowns made of zirconium dioxide are still structural novelties in the permanent prosthetic restorations. Therefore, some aspects of the modeling in dental problems could be analyzed slightly differently from an engineering point of view, introducing the numerical analysis and homogenization hypothesis (cf. Subclauses 3–5). (ii) The crowns located on the single molar and prosthetic bridge are approximated by the simple engineering model, which is significantly more simplethan other numerical models and, consequently, more efficient. The vertical loads are diversified, which can simulate the crushing, mastication, and clenching. (iii) The FE mesh is created and optimized individually for the single crown and prosthetic bridge using different mesh systems, as well as topological and smoothing procedures. The prosthetic bridge is additionally supported by the corresponding part of the gum; the model introduces the elastic support of the molar and the response of the base. (iv) The analysis introduces the tooth caries represented by the change of material parameters inside the predefined volume of the molar. This kind of analysis was not found in the available sources.

## 2. Material Parameters and Loads of Crowns

To determine the numerical model, it is necessary to define the material parameters and loads applied to the prosthetic crown. The problem is difficult because the material and dynamic parameters are individually oriented, and they depend on the hygiene of the oral cavity. Thus, the parameters introduced below are determined statistically and representative for the teeth and prosthetic crowns.

The modulus of elasticity and the tensile and compressive strength of the molars that are subjected to the three-dimensional simulation of mastication and clamping are presented in Table 1 [18]. The enamel is the most load-exposed part of the tooth. The dominant load is the compression: the compressive strength is 37.28 times greater than the tensile strength of enamel. The compressive strength of enamel is only 1.29 times greater than the same parameter of dentin. The material parameters of zirconium dioxide are considerably greater than the parameters for the most resistant part of the tooth (the enamel). The modulus of elasticity is 2.34 times greater, the tensile strength 17.39 times, whereas the compressive strength 2.5 times. Only the Poisson’s ratio is slightly lower (0.58 for the enamel), which is typical for ceramic materials.

According to [16], we can conclude that the most loaded teeth are the molars. The force of mastication for molars is equal to (400–800) N; the premolars, (220–450) N; canines, (130–330) N; and incisors, (90–150) N. The maximal force of mastication is in molars 1.78 times greater than in premolars; 2.42 times than in canines; and 5.33 times than in incisors.

The discussed problems are typical for the strength of materials. Thus, the state variable is the stress defined by the von Mises stress and maximal principal stress. The problem is solved and visualized by means of the ANSYS program.

The material parameters are used to homogenize the material of molar support (Table 2). According to the available sources, gum tissue is characterized by a wide range of strength parameters. Thus, we have introduced two corresponding values (i.e., the minimal and the maximal values of gum tissue); the supporting structure is homogenized alternatively.

The above analysis shows that the most loaded teeth are the molars, and the dominant load is the compressive stress. Therefore, we have to analyze the distribution of stresses and displacements in the prosthetic crown located on the molars, which is loaded by different variants of compressive load.

## 3. Approximation by the Finite Element Mesh

The roots inside the jaw transfer the loads in different directions, particularly horizontally, caused by the tongue and food. Let us assume that the crowns are subjected to vertical and near-vertical loads, which significantly minimize the influence of roots.

Each person is characterized by the individual shape of teeth and their changes; for example, cavities. To analyze the problem unequivocally, the external shape of the model is similar to the molar. Both contain the essential molar elements, i.e., the central groove and functional cusps. The simple model of the prosthetic crown on the mandibular molar was introduced by Dejak [17]. The caries inside the tooth were assumed in the form of a regular cylindrical shape.

The main difficulty is to approximate the prosthetic crown using the appropriate mesh of finite elements. Theoretically, the hexagonal mesh is better for determining the simple shape of the crown and securing the sufficient quality of calculations for the single molar. Thus, the basics are the hexagonal elements. Approximating the prosthetic bridge on two molars, the considerable number of elements in the hexagonal mesh represent the weak quality. The values of the parameter *Skewness* for some elements are highly unfavorable; the mesh is not sufficient and can influence the maps of stresses fields, as well as the symmetry of stress/displacement distributions. On the other hand, the number of elements representing the good qualities is relatively insignificant.

The mesh can be improved by introducing additional procedures. The procedure *Multizone* is based on the block technology, and allows to create the separate mesh areas with the structured and/or unstructured partition, as required. The procedure *Virtual Topology* can improve the mesh using some additional elements like such as edges, points, surfaces, etc. These virtual elements allow creating the irregular topology, and keeping the control over the global shape of the molar by local mesh disturbances. The shape of elements can be defined by the sub-procedures *All Quad*, *Quad/Tri*, and *All Tri*. Some elements can be additionally smoothed using the procedure *Edge sizing* by the division of selected edges on 50 elements and arches on 5 elements. Moreover, the procedure *Check Mesh Quality* and the sub-procedure *Error Limits/Aggressive Mechanical* are used to collect the necessary information about the FE Mesh.

**Figure 1:**
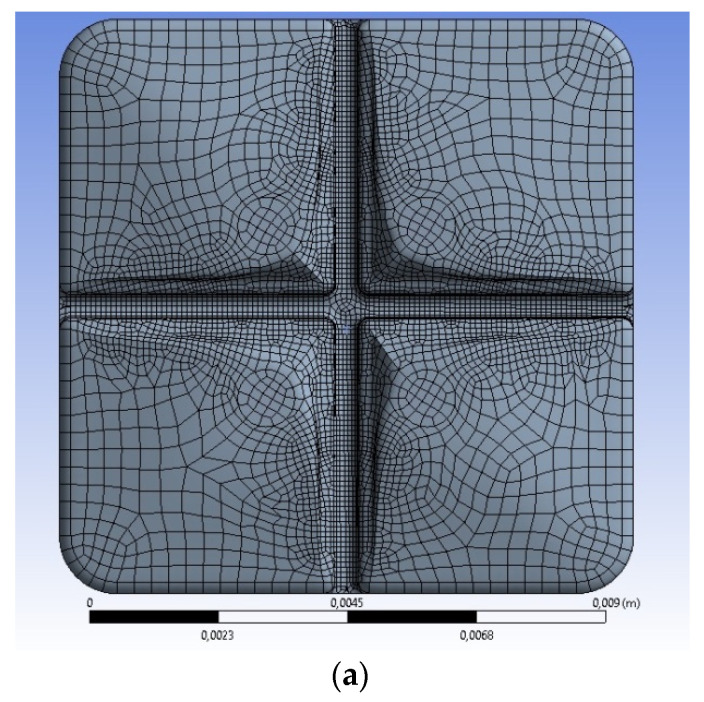

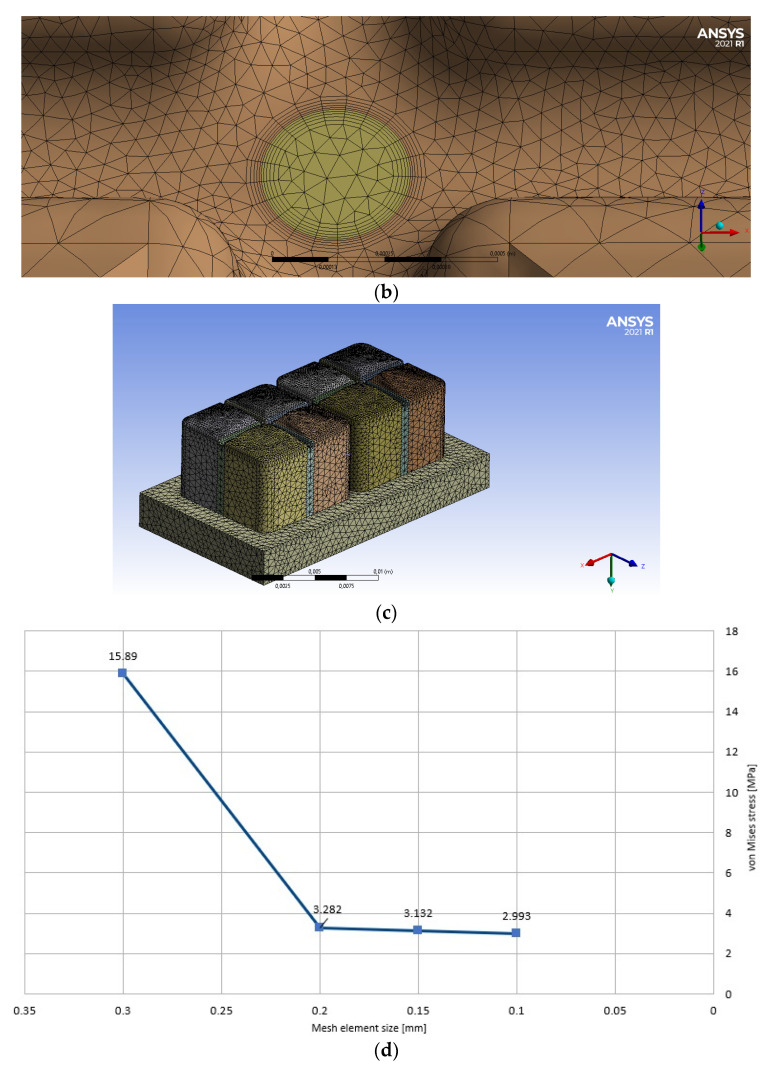

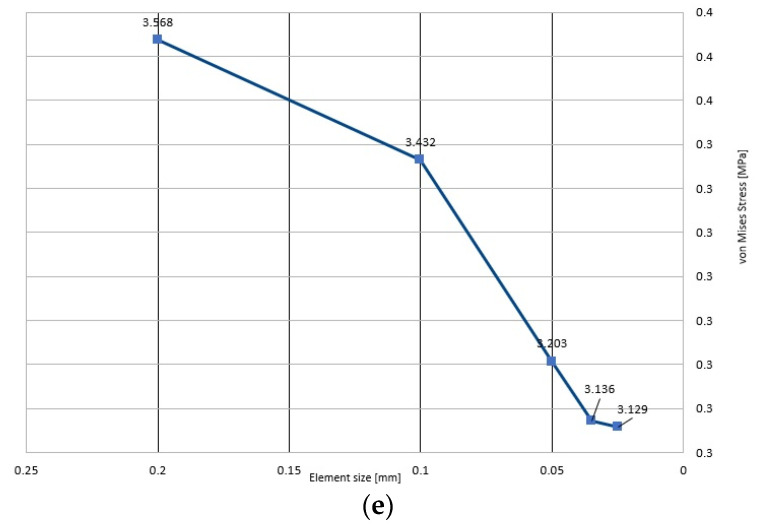
Finite element mesh applied during calculations; (**a**) single prosthetic crown on single molar without caries; (**b**) single prosthetic crown, mesh in the area of caries; (**c**) prosthetic bridge on two molars; (**d**) sensitivity of FE mesh for the single crown (the non-uniform continuous load distributed asymmetrically; linear change from 1 MPa to 0); (**e**) sensitivity of FE mesh for the prosthetic bridge (the non-uniform continuous load distributed asymmetrically; linear change from 1 MPa to 0).

Let us first define the hexagonal mesh for the single molar, Figure 1a. The parameter *Skewness* determines the asymmetry measure as the difference between the current and the ideal shape. The smaller the value, the better the mesh. The recommended value should not be greater than 0.25; the maximal acceptable level during engineering calculations is 0.95. The elements of the quality greater than 0.95 can generate incorrect solutions. In case of a tetragonal mesh, the negligible number of these elements is located far from crucial areas of crown, and does not influence the solutions. The parameter *Orthogonal Quality* defines the orthogonality deviation between the analyzed elements that is the angles ratio using the vectors normal to the centroid. The expected value of *Orthogonal Quality* should be greater than 0.7.

The final number of elements inside the hexagonal mesh is equal to 61,146; the number of nodes 225,652. The obtained mesh is characterized by satisfactory parameters; the single element has an average quality equal to 0.86 (the best is 1, the worst is 0); the standard deviation is 0.27451.

Let us assume that the caries have the regular circular shape of a diameter of 0.33 mm and a length of 1.5 mm. The volume is approximated automatically using the hexagonal mesh of the regular dimensions equal to 0.05 mm without additional procedures (Figure 1b). To determine the changes on the contact surface between the caries and the crown, the inflationary layers are applied. These layers were introduced to ensure the minimal dimensions of the mesh elements at the interface. The increased number of mesh elements is because the dimensions of an element are negligible and the stresses inside approach infinity. Therefore, the mesh approximating the caries is stable during calculations. Irrespective of the problem, the mesh of the prosthetic crown with caries is represented by the same acceptable values of *Skewness* and *Orthogonal Quality* as the basic mesh without the caries. The global mesh is defined by an average quality equal to 0.761 and a standard deviation of 0.12855. The parameters are acceptable, particularly for the complex mesh with two types of material.

**Figure 2:**
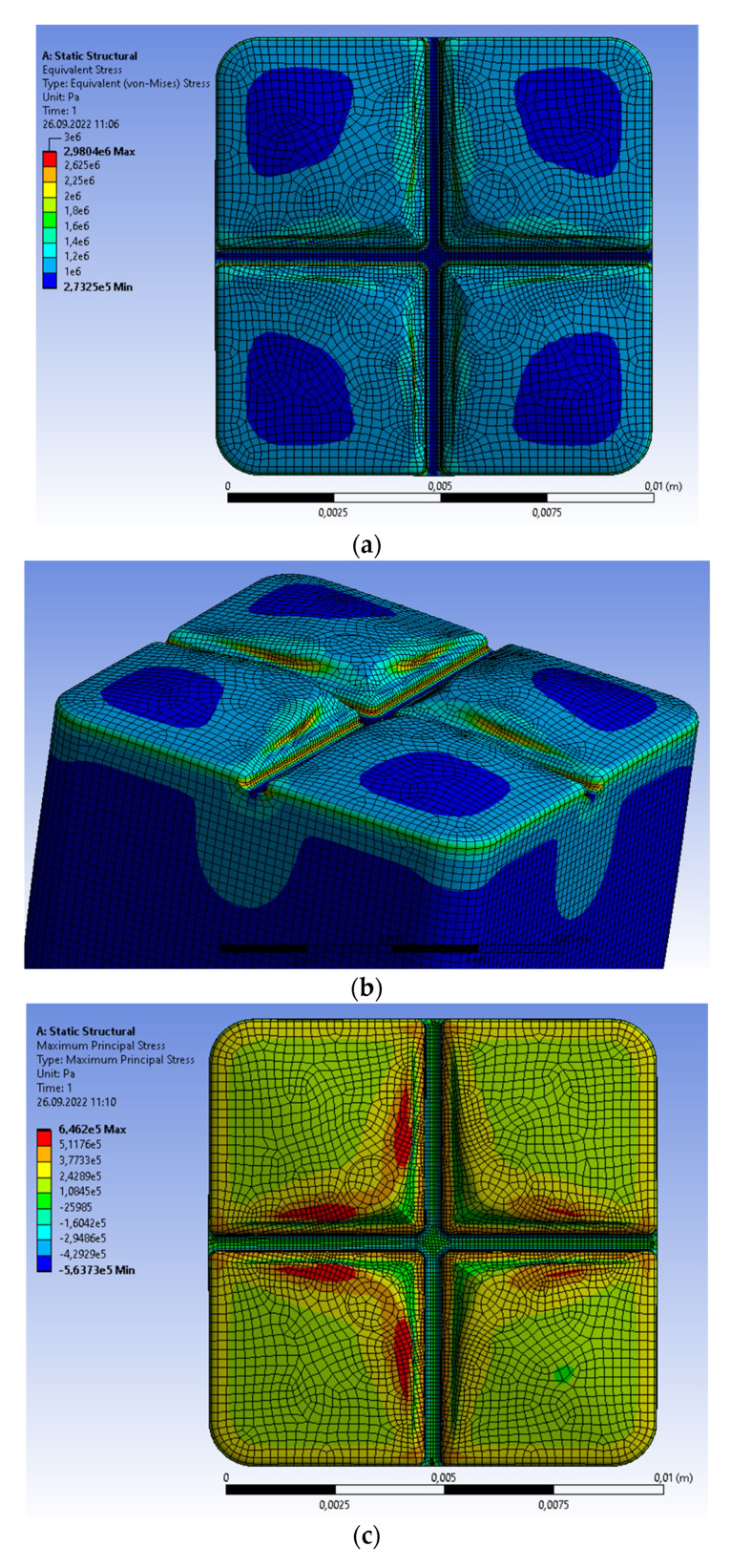

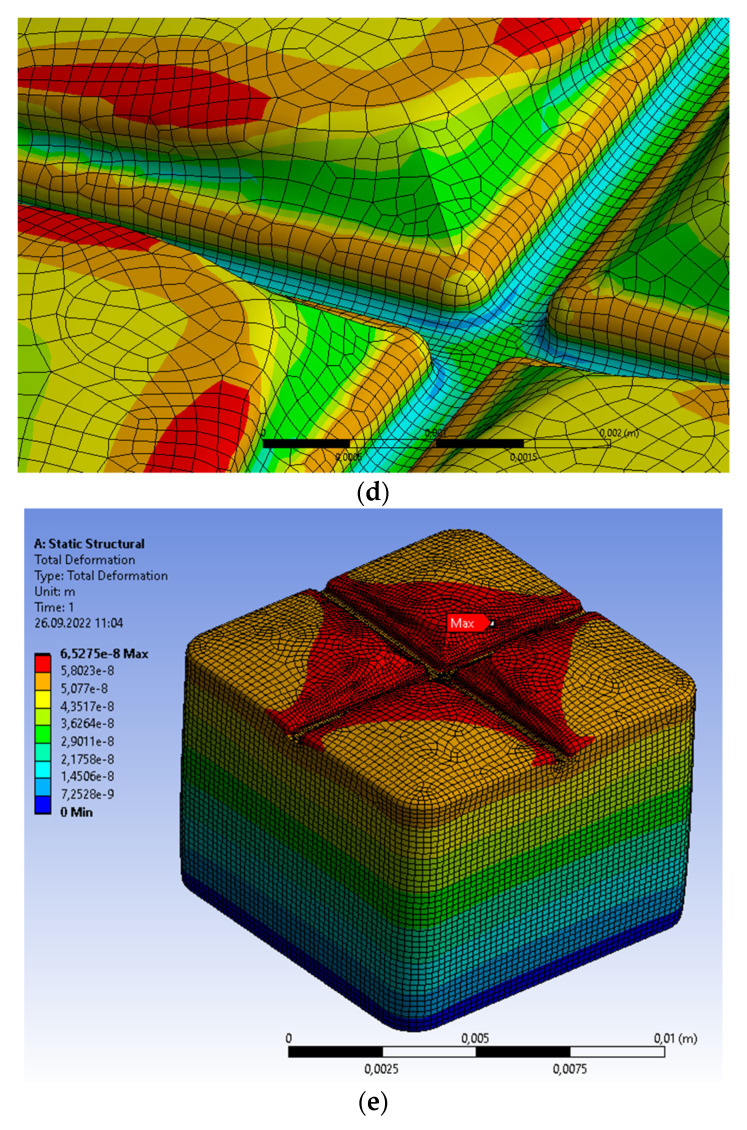
Single prosthetic crown subjected to vertical force of 100 N distributed as continuous load on the upper surface. (**a**,**b**) Areas of maximal von Mises stresses; (**c**) distribution of maximal principal stresses; (**d**) areas of maximal principal stresses; (**e**) distribution of material displacement.

Some specific details of the FE mesh are also shown in the subsequent figures (for example, Figure 2b–d).

In the case of the prosthetic bridge on two molars, it is advantageous to apply a tetragonal mesh, see Figure 1c. The basic mesh is composed of tetragonal elements. The model of a prosthetic bridge is composed of different parts. The upper part is a bridge, which is located on molars, and the lower part approximates a part of the gum which directly contacts the molars. The mesh is created by means of the procedure, *Patch Conforming*, i.e., the model geometry and their crucial points. The dimension of the particular element is equal to 0.2 mm; the initial dimension of elements inside the central groove on the upper surface is 0.05 mm. The tetragonal mesh is compiled from the smoothed elements of the medium smoothing grade.

The bridge on two molars approximates the elastic gum support. The molar base is a homogeneous elastic structure of the material parameters corresponding to the homogenized human tissue; that is, the jawbone and the soft tissue of the prescribed volume and material participations. The final number of mesh elements and the number of nodes are shown in Table 3. The created mesh is satisfactory for engineering calculations because the average quality is equal to 0.756/0.734 (the best is 1, the worst is 0); the standard deviation is 0.13371. The average quality is slightly reduced compared to a single molar of automatically generated mesh, but the standard deviation is slightly greater.

Summarizing, the global quality parameters of hexagonal mesh are good for the simple profile of a crown on a single molar. The complex shape of the prosthetic bridge on two molars should be approximated using the tetragonal mesh. The basic parameters of the FE mesh applied during approximations are summarized in Table 3.

In the case of a single crown (Figure 1d), the change of element mesh size (0.1 mm/0.15 mm/0.2 mm) causes the comparable and negligible increase of von Mises stresses. The corresponding growth from 0.2 mm to 0.3 mm generates a significant drop of stresses; the FE mesh of the model is very coarse, and its quality does not secure the results. The bridge has the more regular increase in stress of the near-asymptotic character (Figure 1e). The change of mesh size (0.025 mm/0.035 mm) causes the negligible increase of von Mises stress equal to 0.007 MPa. The consecutive change of element mesh size (0.035 mm/0.05 mm/0.1 mm) creates the linear growth of stresses. The final increment of mesh (0.1 mm/0.2 mm) generates the decreased drop of stresses equal to 0.136 MPa. The reason is the non-uniform vertical pressure acting on the molar. Its value changes linearly from the maximal 1 MPa on the first molar to 0 on the second. Thus, the near-regular mesh on the part of first molar is subjected to the maximal stresses. The corresponding von Mises stresses are slightly less sensitive to mesh size in this range of dimensions.

Two measures of stresses were introduced because some problems are characterized by different distributions of the principal and von Mises stresses. The principal stress is the real stress inside the molar, which represents the maximum and minimum of normal stresses on a principal plane at a condition of negligible shear stress. The Mises stress is a measure of energy acing on the body, which is calculated using a special formula, and is associated with the yielding criterion of a ductile material. The principal stress is a parameter of theory applicable to brittle materials, whereas the Mises stress is for ductile materials. Generally, the Mises stress determines the fracture limit inside the ductile body. Thus, the values of principal and Mises stresses determine two different aspects of the behavior of a structure subjected to mastication and clenching and the design limitations.

The boundary and loading conditions assumed during the calculations are listed in Table 4. The loads were always time-independent, which determines the steady problem.

## 4. Analysis of Single Prosthetic Crown on Single Molar

The single prosthetic crown is subjected to alternative loads acting vertically as a model of mastication and clenching. Let us first assume that the structure is subjected to clenching. Introducing the uniform distribution, the clenching force equal to 100 N [15,17,18] is assumedas the continuous vertical load on the upper surface of the crown. The fields of stresses and displacements in the prosthetic crown are shown in Figure 2. The von Mises stresses have a diversified distribution (Figure 2a), which is caused by the complex geometry of the upper surface (the functional cusps). The maximal values are determined at the central groove. The elevated values of von Mises stresses are also visible on the groove surfaces, which is demonstrated from a different perspective in Figure 2b. Moreover, Figure 2b illustrates the FE mesh in these regions of the crown, i.e., the areas of global maximum and complex geometry. The maximal principal stresses are determined on the functional cusps, which follows from its curvature (see Figure 2c,d). The maximal values of principal stresses are defined inside the material, whereas von Mises stresses are on the external surface. Thus, the principal stresses indicate a different location of the maximum values on the molar surface than von Mises stresses. The maximal values of the principal stresses are much smaller. The maximal von Mises stresses (2.9804 MPa) and principal stresses (0.6462 MPa) are considerably lower than the compressive strength of enamel, dentin, periodontium, and zirconium dioxide. The distribution of material deformations results from the diversified geometry of functional cusps, Figure 2e. The maximal value of displacement is equal to 6.5275 × 10^−8^ m, and is located on the highest part of cusps. The higher the functional cusps of the molar, the bigger the material deformation, which is a typical material response.

**Figure 3:**
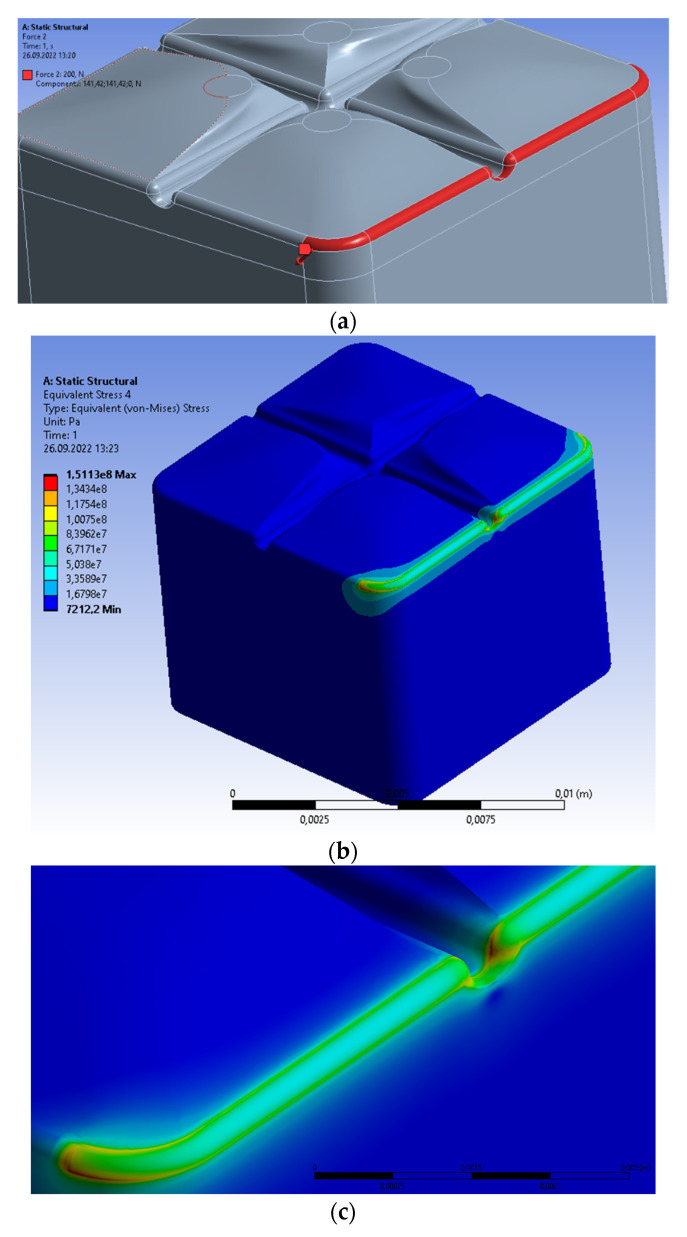

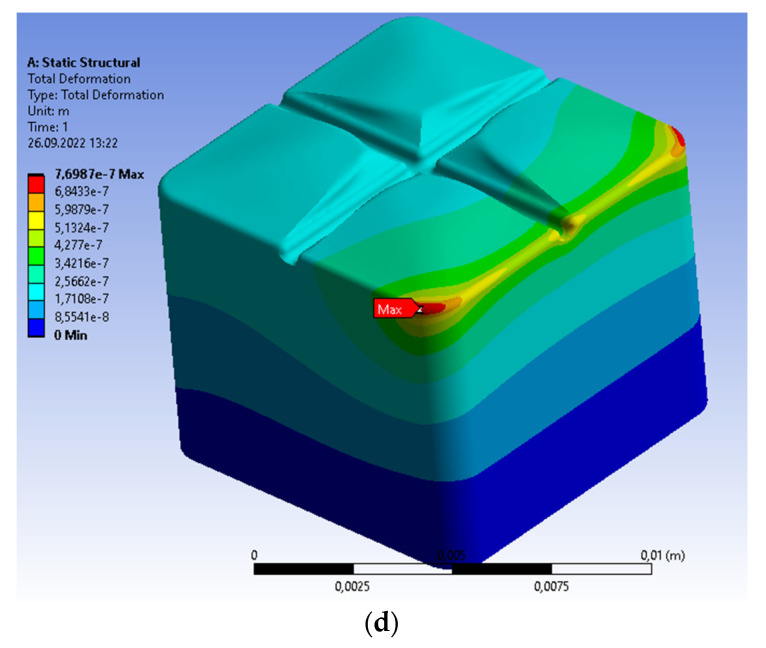
Single prosthetic crown subjected to vertical force of 200 N distributed along the molar edge. (**a**) Force on the edge of molar; (**b**) general view of von Mises stresses; (**c**) area of maximal von Mises stresses inside the curvature; (**d**) distribution of material displacement.

The next analyzed case is the crown subjected to the assumed force of 200 N [15] acting vertically at the edge (Figure 3a). The force generates a linear distribution of load intensity along the edge in respect to the insignificant surface of the acting load. The general view of von Mises stresses presents Figure 3b, whereas the distribution in the central part shows Figure 3c. The maximal stresses are located inside the central groove at its maximal curvature. The maximal value equal to 151.1300 MPa does not exceed the compressive strength for enamel, dentin, periodontium, and zirconium dioxide, although the force is applied on the infinitesimal surface. The maximal displacements are determined at the loaded edge, but the deformation is more regular than the distribution of stresses. The reason is the continuous material and relatively small area of external loads. The maximal displacement is equal to 7.6987 × 10^−7^ m, and is greater than determined previously, i.e., for the case of the stresses uniformly distributed on the tooth surface.

**Figure 4:**
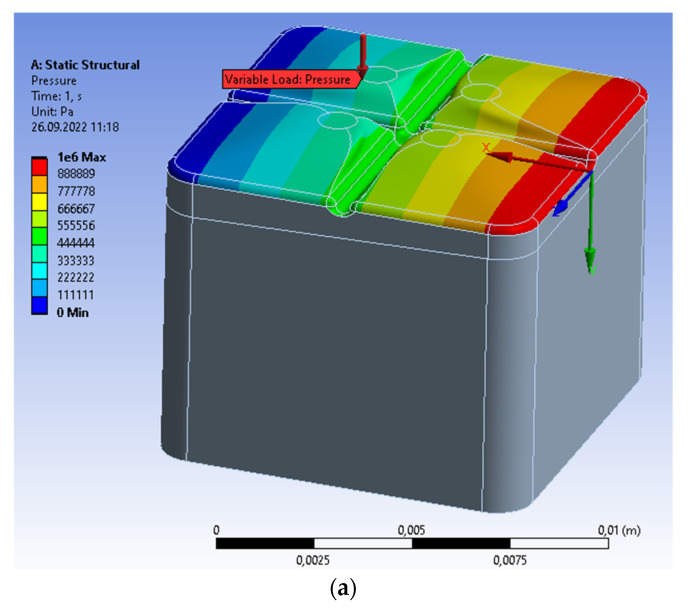

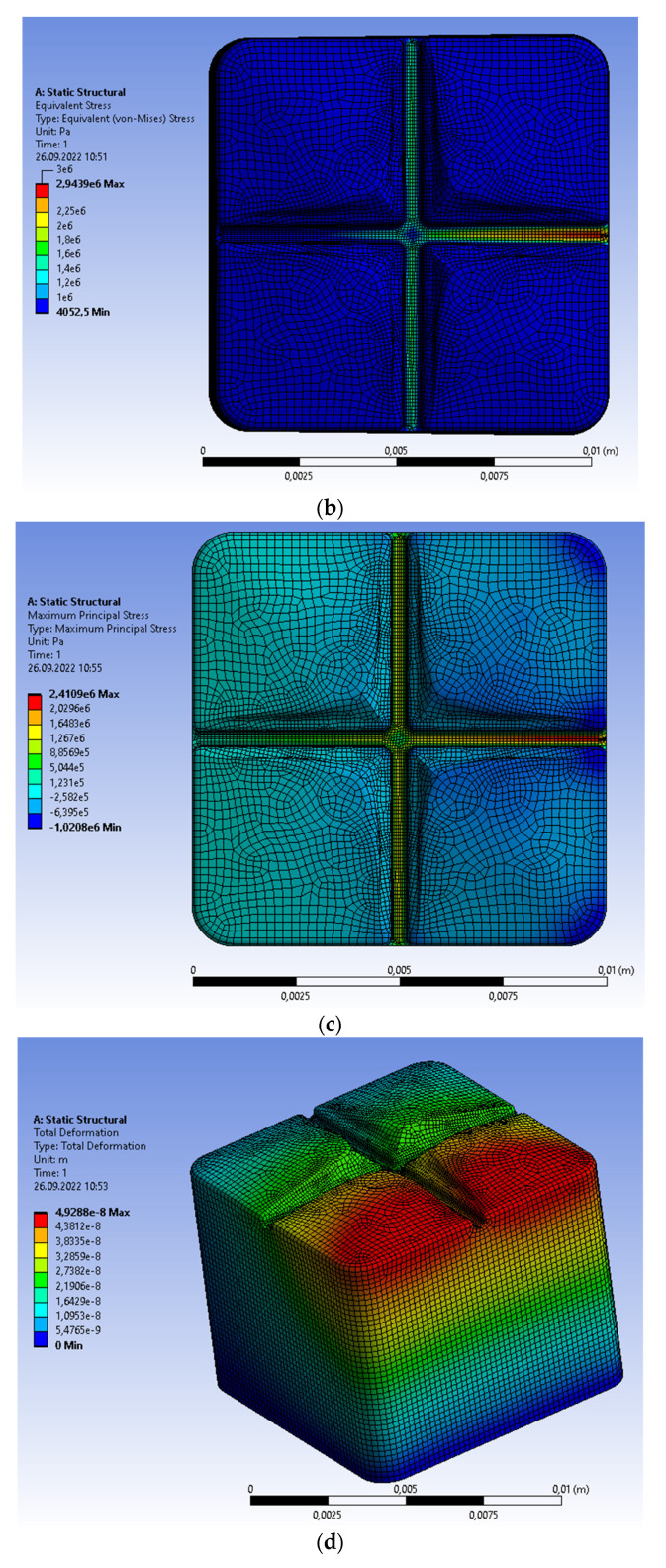
Single prosthetic crown subjected to the continuous vertical load; the linear variability from 1 MPa to 0. (**a**) Discretization of load on the upper surface; (**b**) distribution of von Mises stresses; (**c**) distribution of maximal principal stresses; (**d**) distribution of material displacement.

The crown on a single molar can be alternatively subjected to crushing, i.e., the non-uniform continuous load distributed asymmetrically. Let us assume that the vertical pressure changes linearly along the molar from the maximal value of 1 MPa to 0. The maximal vertical pressure, 1 MPa, results from the maximal force of tooth clamping that is equal to 500 N [15] and distributed over an area of 10^−4^ m^2^ (i.e., the approximate orthogonal projection of the molar on the horizontal surface). To satisfy the computational requirements, it is necessary to distribute a uniformly variable load into the rectangles of a constant stress value (Figure 4a). The maximal values of von Mises stresses are determined at the bottom part of the central groove (Figure 4b). The stresses change according to the applied load from the maximal value to 0. The principal stresses have a similar form (Figure 4c). The maximal value of the von Mises stresses is equal to 2.9439 MPa; the principal stresses are 2.4109 MPa; both are below the compressive strength for dentin equal to 297 MPa (i.e., the minimal in Table 1). The asymmetric distribution of stresses generates the asymmetric displacements of the maximal value equal to 4.9288 × 10^−8^ m (Figure 4d).

**Figure 5:**
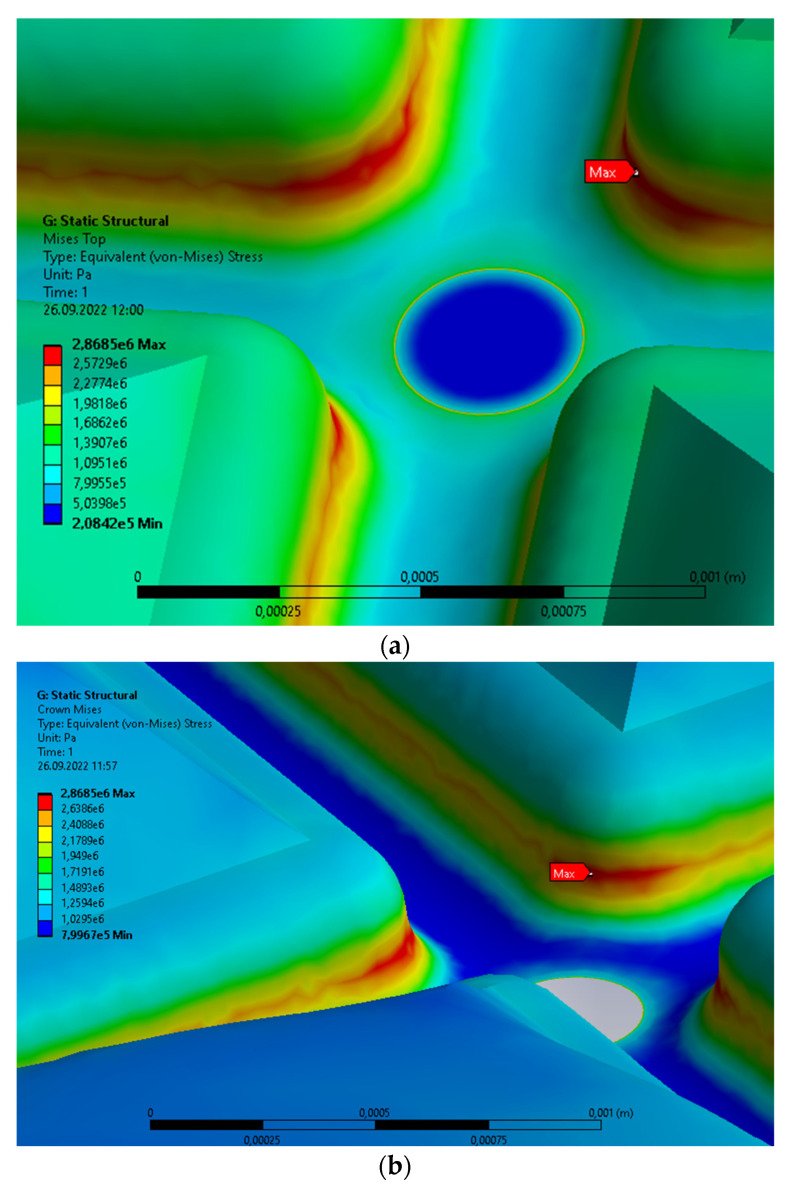

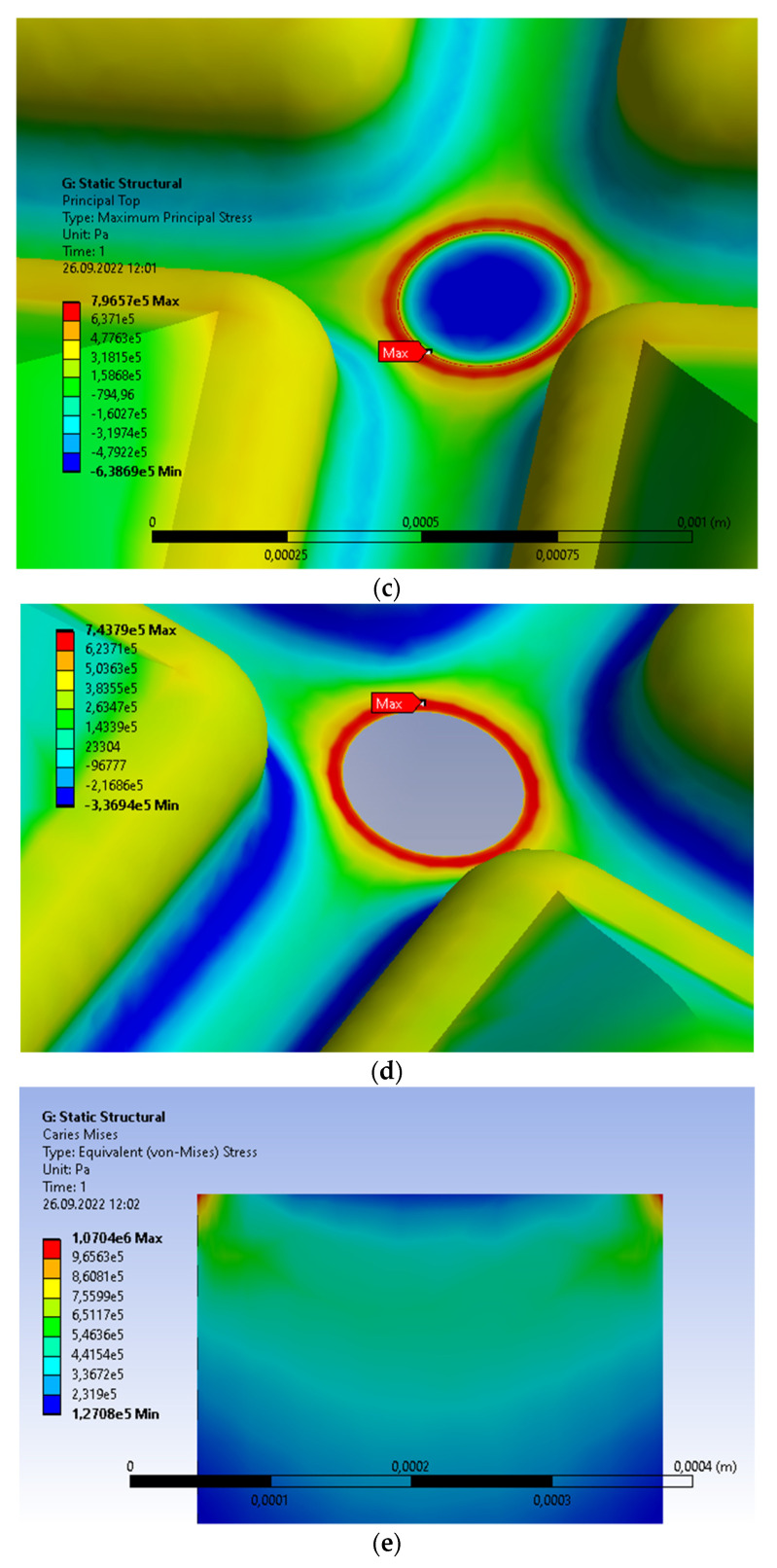

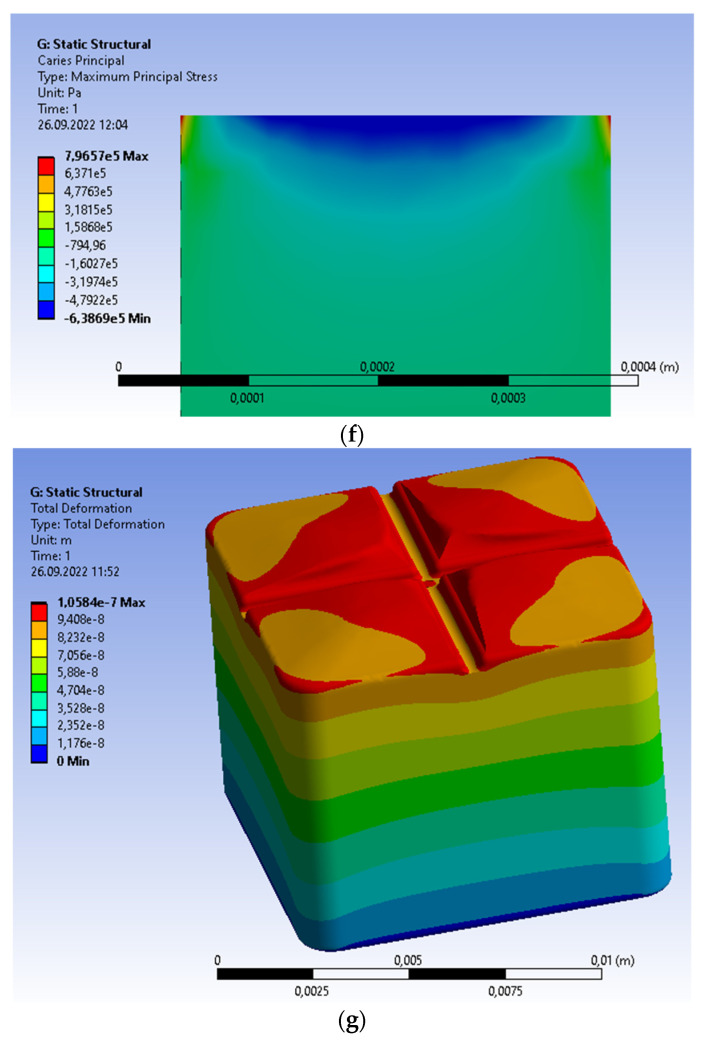
Single prosthetic crown subjected to the continuous vertical load with caries; the linear change from 1MPa to 0. Distribution of the following parameters: (**a**) area of maximal von Mises stresses with the volume filled by caries; (**b**) area of maximal von Mises stresses with the void volume of caries; (**c**) area of maximal principal stresses with the volume filled by caries; (**d**) area of maximal principal stresses with the void volume of caries; (**e**) area of maximal von Mises stresses inside the volume filled by caries; (**f**) maximum principal stresses inside the volume filled by caries; (**g**) displacement.

Let us consider the crown on a single molar with caries: Figure 5 compared with Figure 2. The vertical clenching force, equal to 100 N [15,17,18], is distributed uniformly on the upper surface. The caries have a regular cylindrical shape, and the material properties are listed in Table 2. The material characteristics inside the caries volume are considerably lower than the other materials of the molar and crown. The more advanced the caries, the lower the value of these parameters. Theoretically, the lower limit of material parameters is the negligible value that corresponds to the empty volume of caries material. However, the analysis is more complicated than in previous cases. The distribution of von Mises stresses for the volume filled by caries is shown Figure 5a, and the void in Figure 5b. The maximal values of stresses are equal to 2.8685 MPa in both cases. Thus, both are comparable, but the second is smaller with regard to the negligible material parameters. The maxima are located in the central part of crown at the central groove. The maximal principal stresses for the volume filled by caries are presented in Figure 5c (0.79657 MPa), whereas the void is shown in Figure 5d (0.74379 MPa). Generally, the values are smaller than the values obtained for the molar without caries. The second value is the smallest of all that were obtained during both analyses. The stresses distributions are different than the corresponding distributions for the von Mises stresses. The maxima are located at the contact surface with caries, which results from the diversified material characteristics, and it is typical for the principal stresses. The corresponding stresses inside caries are equal to 1.0704 MPa (Figure 5e; the von Mises stress); 0.79657 MPa (Figure 5f; the maximal principal stress). The main reason of this situation is the weak material inside the caries volume. The maximal displacement of 1.0584 × 10^−7^ m is considerably larger than the same parameter of the molar without caries.

**Figure 6:**
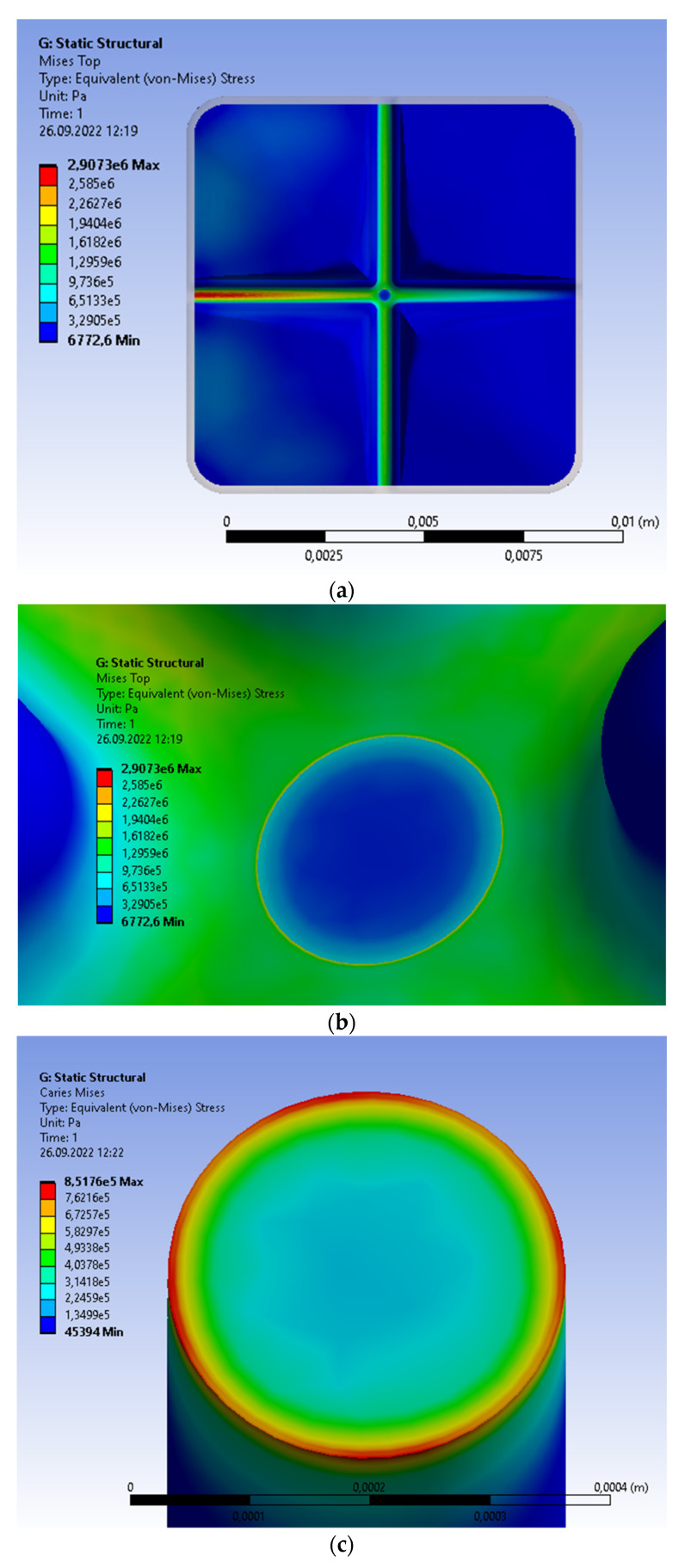

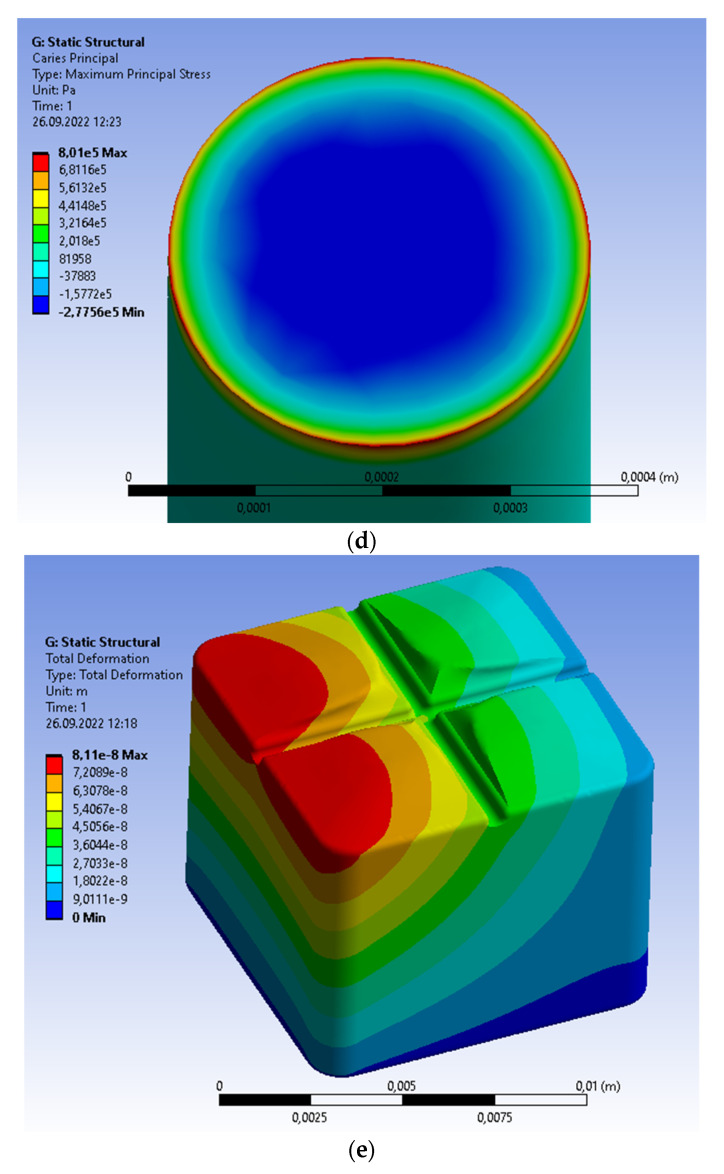
Single prosthetic crown subjected to the continuous vertical load with caries; the linear change from 1 MPa to 0. Distribution of the following parameters: (**a**) von Mises stresses for the crown; (**b**) area of maximal von Mises stresses in the crown area contacting caries; (**c**) von Mises stresses inside the volume filled by caries; (**d**) area of maximal principal stresses inside the volume filled by caries; (**e**) material displacement.

The single molar with caries can be subjected to the non-uniform continuous load; see Figure 6 compared with Figure 4. The vertical pressure changes linearly from the maximal value of 1 MPa to 0. To apply the variable load, we introduce the defined number of rectangles (in this case, nine rectangles) subjected to uniform load; the resultant force on the single surface is the same for the constant and variable load (Figure 4a). The von Mises stresses for the crown have a distribution similar to those obtained without caries; the maximal value is located at the bottom part of groove and is equal to 2.9073 MPa (Figure 6a,b). The volume with caries is characterized by weak material parameters (Table 2). The maxima are located at the contact surface caries/crown, and for the von Mises stresses equal to 8.5176 MPa (Figure 6c); the maximal principal stress is 0.80100 MPa (Figure 6d). The obtained values are insignificant in respect to the compressive strength for dentin equal to 297 MPa (the minimal are in Table 1). The displacement is asymmetric of the maximal value equal to 8.11 × 10^−8^ m (Figure 6e). This means that the displacement for the molar with caries is 1.65 times greater than the displacement without caries. The reason is the reduced level of material parameters, despite the small volume of caries/void space.

Summarizing, the obtained fields of the von Mises stresses and principal stresses depend on the distribution and value of the external load. Irrespective of the analyzed crown and applied load, the maximal values do not exceed the compressive strength for enamel, dentin, periodontium, and zirconium dioxide. The same situation is determined in extreme cases; that is, either the loads acting at the edge of crown or those considerably greater than expected (1 MPa).

## 5. Analysis of Prosthetic Bridge on Two Molars

The obtained values of maximal stresses for the single crown are always below the compressive strength of all materials listed in Table 1. Therefore, the analysis of the double prosthetic bridge is focused on the sensitivity of maximal stresses and deformations to elastic support by the gum/jaw structure.

**Figure 7:**
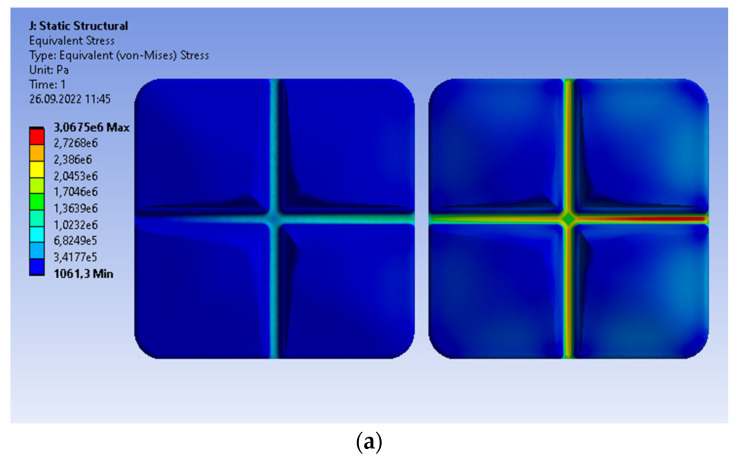

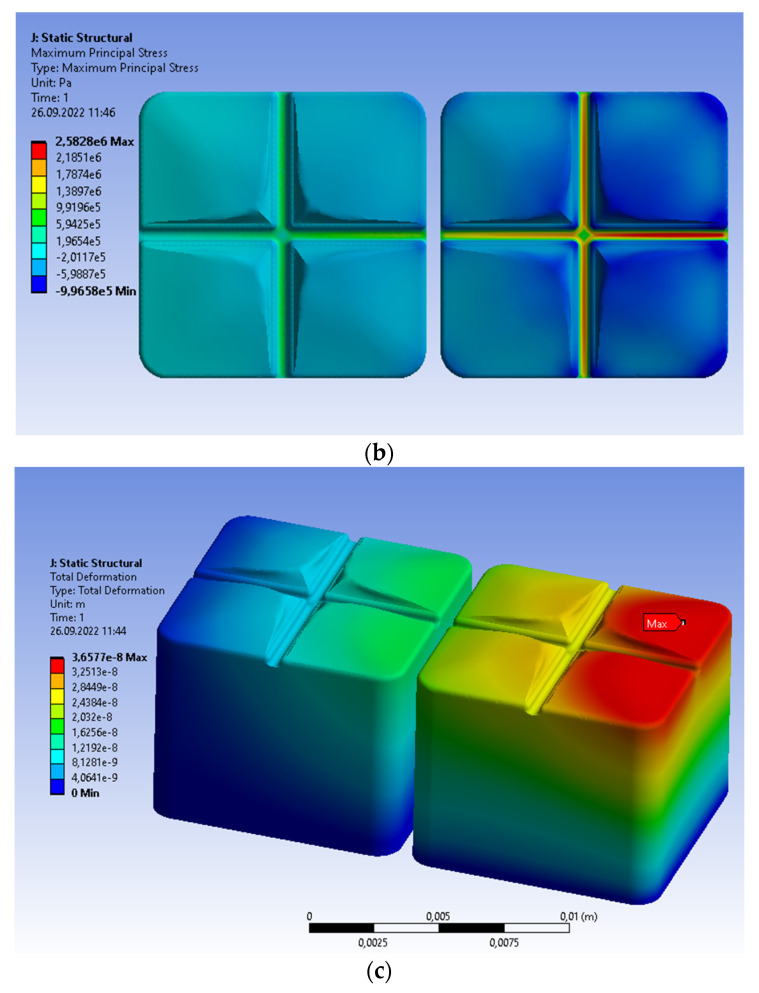
Prosthetic bridge subjected to the continuous vertical load; the linear change from 1 MPa to 0; the rigid support. Distribution of the following parameters: (**a**) von Mises stresses; (**b**) maximum principal stresses; (**c**) material displacement.

Let us create the reference results using the rigid support of molars. The double prosthetic bridge is subjected to the most unfavorable load, i.e., the non-uniform vertical pressure. Its value changes linearly from the maximal 1 MPa on the first molar to 0 on the second. The distribution of the von Mises stresses results from the distribution of loads (Figure 7a). Similarly to the single crown, the maximum stress is determined at the same bottom part of the central groove and is equal to 3.0675 MPa. The area of maximal von Mises stresses covers this single groove. The maximal principal stress is slightly different (Figure 7b). Although the maximum is located at the same point of crown and equal to 2.5828 MPa, the increased stresses are determined in other grooves of the same molar. The obtained values are insignificant compared to the compressive strength of 297 MPa, i.e., the compressive strength for dentin. The global displacement of the maximal value equal to 3.6577 × 10^−8^ m is caused by the diversified distribution of loads (Figure 7c). The maximal values of the von Mises stresses, principal stresses, and displacement are greater than the corresponding values inside the single crown.

To analyze the influence of the elastic base, we consider the double prosthetic bridge with the elastic gum as a support of the molar. The corresponding material parameters of gums, caries, and zirconia are listed in Table 2. The model has a geometrical shape similar to the real gum, but without the roots of the complicated geometry. The influence of the roots and jaw are not deciding factors in the case of vertical loads. The roots/jaw system is now substituted by the homogeneous elastic structure of the material parameters corresponding to the homogenized human tissue: the jawbone and the soft gum tissue of the prescribed volume and material participations (Table 2). In fact, the previous prosthetic crowns were analyzed on the rigid base, and the maxima were determined in the most unfavorable conditions.

**Figure 8:**
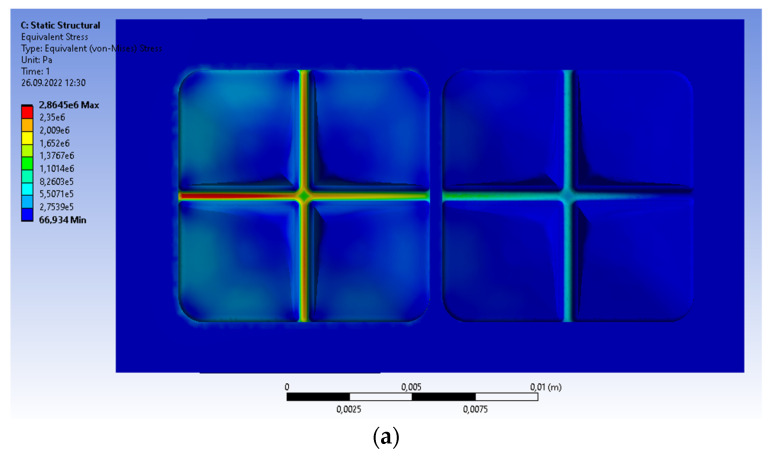

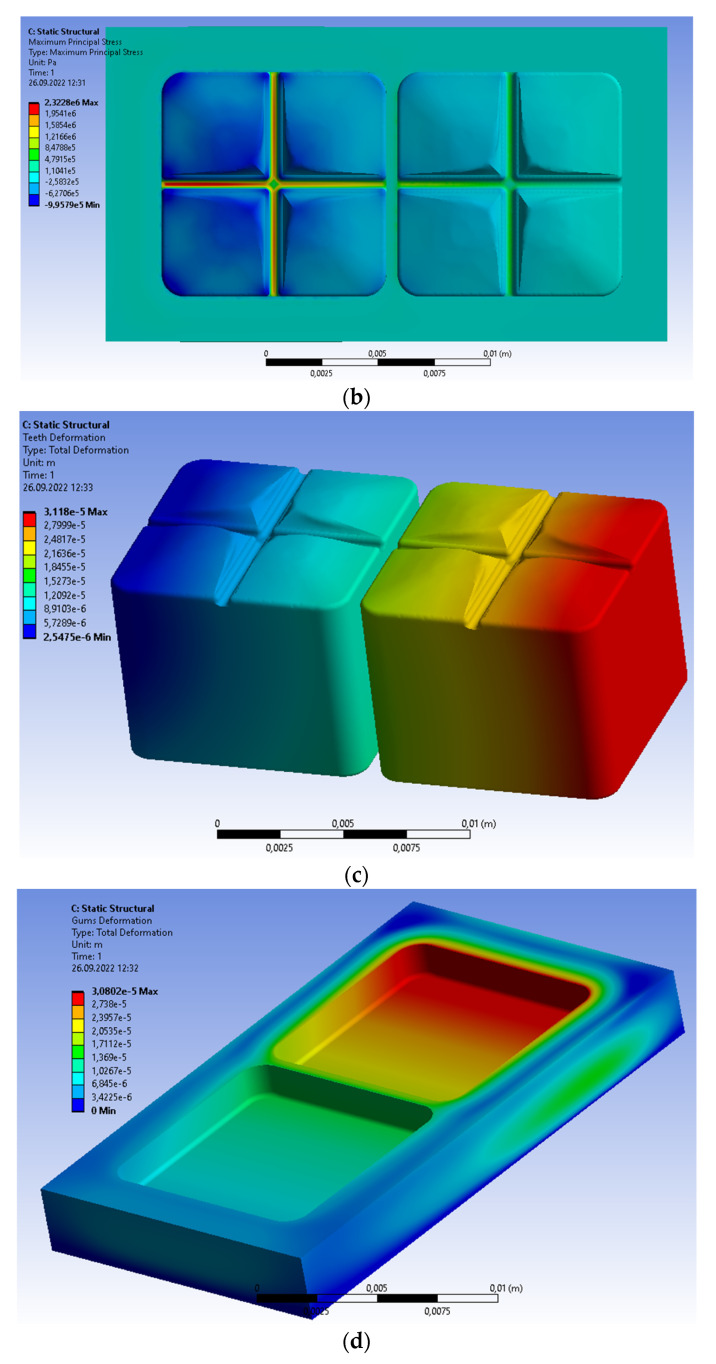
Prosthetic bridge subjected to the continuous vertical load; the linear change from 1 MPa to 0; the elastic support. Distribution of the following parameters: (**a**) von Mises stresses; (**b**) maximum principal stresses; (**c**) teeth deformation; (**d**) support displacement.

The distributions of all parameters are similar to the rigidly supported bridge, but the values are slightly different. The von Mises stresses are considerably smaller than before (the elastic support, 2.8645 MPa; the rigid, 3.0675 MPa); the maximum is at the same area (Figure 8a). The maximal value of principal stress is equal now to 2.3228 MPa; for the rigid support, 2.5828 MPa (Figure 8b). The displacement of the double bridge with the elastic support is much greater than before (3.1180 × 10^−5^ m related to 3.6577 × 10^−8^ m, Figure 8c). The elastic support is also deformed, and the maximal displacement is comparable with the displacement of the bridge (3.0902 × 10^−5^ m, Figure 8d). The displacements are asymmetric according to transferred load.

Thus, the analyzed stresses and displacement are sensitive to elastic support. The differences between stresses are relatively insignificant, but the displacement is considerably higher than the value obtained for the rigid gum. Moreover, the applied load generates the displacements of both the prosthetic bridge and elastic support, which is a typical material response of the deformable structure located on the deformable base. The stresses/displacements are transferred as the dynamic impulse through the gum to the jaw and, finally, the vertebral column. However, this problem is beyond the scope of the paper presented.

## 6. Comparison with the Results from Literature

The results of the numerical analysis being consistent with the data in the available literature may be a preliminary confirmation of the homogenization hypothesis (cf. Table 5). It is easy to see that the loading conditions are similar, but not identical. Thus, we can compare the order of magnitude of the loads determined during the mentioned analyses.

The similar structure of the crown, the external geometrical profile of the molar, and the loading conditions were analyzed by Dejak [17]. The only difference is the variable load and its operation surface during the clenching. The maximal pressure was applied on functional cusps, i.e., it is reduced to the limited areas. Regardless of the differences, the obtained von Mises stresses are comparable to the results obtained by other researchers. The maximal values according to [17] were between 1.27 MPa and 8.206 MPa (depending on the clenching phase), whereas in the presented paper, it is 2.9804 MPa (cf. Figure 2). The force assumed during our calculations is equal to 100 N, which is a typical reference value from the literature. The concentrated load is uniformly distributed on the upper surface of the molar.

The crown with overhang located on the molar was analyzed by Materac and Niesłony [15]. Mastication and clenching were simulated using the vertical load in the form of a concentrated force of 200 N acting on the overhang. The results are comparable and are the same order of magnitude. The maximal stresses according to [15] were equal to 241 MPa; in the author’s calculations, they are equal to 142.6900 MPa. The divergence is caused by the concentrated force acting asymmetrically [15], which generatesa bending moment inside the jaw. Thus, an additional factor is now a non-vertical load transferred by means of the roots.

Regardless of the case under consideration, all values obtained did not exceed the tensile strength of dentin that is the lowest permitted value of the molar.

## 7. Conclusions

The stress and displacement fields in prosthetic crowns made of zirconium dioxide were analyzed using a numerical approach of homogenization hypothesis, i.e., for the rigid and the deformable elastic support of the homogenized properties. The hypothesis was formulated for molars subjected to vertical compression, which is the dominant load during mastication and clenching. The material parameters are characteristic for human tissue represented by volume and material participations of the jawbone, and the soft tissue of gum. The distributions of stress and displacement are comparable with the results in the available literature, which can be accepted as a preliminary confirmation. However, it is imperative to prove the hypothesis using other methods.

The rigid support was introduced in the case of a single crown and the elastic support for the prosthetic bridge on two molars. According to numerical simulations, a rigid support results in higher stresses on the molar compared to the stresses for the elastic base. The obtained distributions of the von Mises stresses and principal stresses differ in distributions and maximal values. However, the maximal stresses are insignificant compared to the compressive strength for enamel, dentin, periodontium, and zirconium dioxide; zirconium dioxide is the most resistant material surrounding the dentine. The strength is satisfactory, even though the load is much higher than expected or concentrated at the molar edge.

In the case of caries in a single molar, the maximal von Mises stresses and principal stresses are located at the contact surface caries/crown. Both values did not exceed the compressive strength for dentin, i.e., the minimal in Table 1. The displacement of the molar with caries was much higher than the displacement without caries. The reason is the reduced level of material parameters despite the small volume of caries.

The prosthetic bridge on two molars was analyzed in respect of the rigid and elastic support. The obtained stresses were comparable in both cases and located at the same areas; the values did not exceed the compressive strength for dentin. The displacements were completely different. The global deformation for the elastic base was a sum of molar and support deformations; the value was considerably greater than that obtained for the rigid support. Therefore, the elastic support transferred a part of the load; the problem is interesting from a dynamic point of view, but beyond the scope of paper presented.

The crown on molars can be optimized, in respect to the shape and material properties, using specific optimization techniques [22,23,24,25]. The obtained results of stresses and displacement fields can be a part of the analysis stage during optimization. The synthesis stage allows to determine the optimal crown subjected to compressive loads [25,26,27,28]. These problems create new opportunities for research and analysis.

## Figures and Tables

**Table 1:** Material parameters of molars subjected to three-dimensional simulation of mastication and clamping [18].

Structure	Modulus of Elasticity, GPa	Poisson’s Ratio	Tensile Strength, MPa	Compressive Strength, MPa
Enamel	84.1	0.33	11.5	384
Dentin	18.6	0.31	105.5	297
Periodontium	0.05	0.45	-	-
Zirconium dioxide ceramics	210	0.19	200	900

**Table 2:** Material parameters of gums, caries, and zirconia.

Parameter	Element				
	Young’s Modulus, MPa	Poisson’s Ratio	Bulk Modulus, MPa	Shear Modulus, MPa	Tensile Yield Strength, MPa
Gums—minimal values	13.55	0.45	45.167	4.6724	2.75
Gums—maximal values	25.95	0.45	86.5	8.9483	5.13
Caries	900	0.33	8823.5	3383.5	1.1
Zirconia	1.38 × 10^5^	0.259	9.5436 × 10^4^	5.4805 × 10^4^	1.32 × 10^2^

**Table 3:** Basic parameters of the FE mesh applied during approximations.

Case	Boundary Condition	Number of Elements	Number of Nodes	Mesh Type	Skewness (Average)	Orthogonal Quality (Average)
Single crown on single molar subjected to clenching	Rigid support	61,146	225,652	Hexahedral, Multizone	0.226	0.860
Single crown on single molar with caries subjected to clenching	Rigid support	444,296	648,857	Tetrahedral, Patch Conforming Method	0.237	0.761
Bridge on two molars subjected to clenching	Rigid support	1,128,366	1,609,630	Tetrahedral, Patch Conforming Method	0.242	0.756
Bridge on two molars subjected to clenching	Elastic support	2,184,324	3,039,131	Tetrahedral, Patch Conforming Method	0.264	0.734

The mesh quality and final number of elements chosen for simulations should be validated with a mesh sensitivity analysis. The distributions of the von Mises stresses versus the mesh element size are shown in Figure 1d,e. The stresses according to the modified von Mises criterion are the basic state variable in the strength of materials. The displacements are too sensitive to the element size; a small change in size generates a significant change in displacement.

**Table 4:** Boundary and initial conditions applied during the analyses.

Case	Boundary Condition	Loads
Single crown on single molar subjected to clenching.	Rigid support	The vertical clenching force equal to 100 N distributed uniformly on the upper surface.
Single crown on single molar subjected to clenching.	Rigid support	The assumed force 200 N acting vertically at the edge; decomposed on the infinitesimal area.
Single crown on single molar subjected to clenching.	Rigid support	The non-uniform continuous load; result of asymmetric distribution of the crushed substance. The vertical pressure changes linearly along the molar from the maximal 1 MPa to 0.
Single crown on single molar with caries subjected to clenching.	Rigid support	The vertical clenching force equal to 100 N distributed uniformly on the upper surface
Single crown on single molar with caries subjected to clenching.	Rigid support	The non-uniform continuous load; result of asymmetric distribution of the crushed substance. The vertical pressure changes linearly along the molar from the maximal 1 MPa to 0.
Bridge on two molars subjected to clenching.	Rigid support	The non-uniform continuous load; result of asymmetric distribution of the crushed substance. The vertical pressure changes linearly along the molar from the maximal 1 MPa to 0.
Bridge on two molars subjected to clenching.	Elastic support	The non-uniform continuous load; result of asymmetric distribution of the crushed substance. The vertical pressure changes linearly along the molar from the maximal 1 MPa to 0.

**Table 5:** Comparison of obtained calculation results in relation to the corresponding results from the available literature.

Source	Description	Measure	Max. Value, MPa
Bramanti et al. [14]	The crown is located centrally, in the upper part of the molar. The load is the oblique concentrated force of the value 412.54 N. The spatial distribution: 65% vertically; 35% horizontally.	The principal stress	24.5
Dejak [17]	The crown is located centrally, in the upper part of the molar. Simulation of mastification and clenching between two opposing maxillary molars with three elastic modules: a carrot, an almond, and a nut. The maximum pressureexerted on functional cusps, i.e., is reduced to the limited areas.	The von Mises stresses	Depending on the food and the clenching phase: between 1.27 MPa and 8.206 MPa
Materac, Niesłony [15]	The crown located on the molar. Simulation of mastification and clenching by the vertical load. The load is the concentrated force of 100 N.	The von Mises stresses	14 MPa
Materac, Niesłony [15]	The crown with overhang located on the molar. Simulation of mastification and clenching by the vertical load. The load is the concentrated force of 200 N acting on the overhang.	The von Mises stresses	241 MPa
Dzięgielewski, Regulska, Korycki, Klimek	The crown is located centrally, in the upper part of the molar. Simulation of mastification and clenching by the vertical load. The load is the force of 100 N decomposed as the continuous vertical load on the upper surface.	The von Mises stresses	2.9804 MPa
		The principal stresses	0.6462 MPa
Dzięgielewski, Regulska, Korycki, Klimek	The crown is located centrally, in the upper part of the molar. Simulation of mastification and clenching by the vertical load. The load is the force of 200 N acting vertically at the edge on the infinitesimal surface.	The von Mises stresses	151.1300 MPa
Dzięgielewski, Regulska, Korycki, Klimek	The crown is located centrally, in the upper part of the molar. Simulation of mastification and clenching by the vertical load. The vertical load changes linearly along the molar from the maximal value 1 MPa to 0.	The von Mises stresses	2.9439 MPa
		The principal stresses	2.4109 MPa

## Data Availability

Not applicable.
